# Quantitative assessment of ticks in a hot cave in the Amazon reveals that the genus *Antricola* (Ixodida: Argasidae) has the highest cave tick population density on planet

**DOI:** 10.1007/s10493-026-01146-7

**Published:** 2026-05-21

**Authors:** Flávio A. Terassini, Marcelo B. Labruna, Sebastián Muñoz-Leal, Luís Marcelo A. Camargo, André de A. R. Aguirre

**Affiliations:** 1 Department of Medicine, São Lucas, Porto Velho, RO, AFYA Brazil; 2 Institute of Biomedical Sciences 5, University of São Paulo, Monte Negro, RO Brazil; 3 Department of Preventive Veterinary Medicine and Animal Health, Faculty of Veterinary Medicine and Animal Science, University of São Paulo (USP), São Paulo, SP Brazil; 4 Department of Animal Science, Faculty of Veterinary Sciences, University of Concepción, Chillán, Chile; 5 Entomology Laboratory, Fiocruz Rondônia, Porto Velho, RO Brazil; 6 Graduate Program in Biodiversity and Biotechnology, BIONORTE Network, Porto Velho, RO Brazil; 7 Centro Universitário UNIFAEMA, Ariquemes, Rondônia Brazil; 8 Laboratório de Medicina Tropical da UFAC, Rio Branco-AC, Brazil; 9 INCT/Conexão-Porto Velho-RO, Porto Velho, Brazil; 10 CEPEM/SESAU-PORTO VELHO-RO, Porto-Velho, Brazil

**Keywords:** Tick abundance, Argasid mites, Bat guano, Amazon rainforest, Brazil

## Abstract

Within Argasidae ticks, the genus *Antricola* is restricted to the New World and exhibits a unique biology: only larvae are hematophagous and parasitize bats, whereas nymphs and adults are free-living and supposedly to feed on bat guano in hot caves with high temperatures (30–38 °C) and relative humidity close to 100%. This study quantified the tick population in an Amazonian hot cave located in the Natural Park of Porto Velho (Rondônia, Brazil), inhabited by thousands of insectivorous bats. Standardized guano samples (100 cm² at 10 points) were collected during dry and rainy seasons and examined in the laboratory. The guano-covered area totaled 146.34 m². A total of 7,388 ticks was collected in the dry season and 8,753 in the rainy season, with more than 99% belonging to *Antricola*, mainly *A. delacruzi*. The estimated mean density was 80,705 ticks/m², corresponding to an approximate population of 11.8 million individuals on the cave floor—the highest tick density ever recorded in a cave worldwide. Only nymphs and adults were found, suggesting that *Antricola* eggs and larvae remain in wall crevices, and that rapid larval development, combined with continuous guano feeding, promotes long post-larval longevity. These findings highlight a highly specialized host–parasite dynamic and emphasize the importance of conserving Amazonian cave ecosystems and surrounding forests, which are threatened by deforestation and other anthropogenic pressures.

**Supplementary Information:**

The online version contains supplementary material available at10.1007/s10493-026-01146-7.

## Introduction

Currently, ticks (Acari: Ixodida) comprise around 1,000 extant species worldwide, which are divided into three families: approximately 770 hard tick species (Ixodidae), around 225 soft tick species (Argasidae), and the monotypic family Nuttalliellidae (Mans et al. 2021; Guglielmone et al. 2023). The Brazilian tick fauna currently comprises 78 valid species categorized into two families: Ixodidae (54 species) and Argasidae (24 species) (Labruna et al. 2024; Tojal et al. 2025).

Argasids (soft ticks) have been traditionally classified into five genera: *Argas*,* Antricola*,* Otobius*,* Nothoaspis* and *Ornithodoros* (Nava et al. 2017). Due to some inconsistencies in this classification, recent phylogenetic studies have proposed the taxon *Antricola* as a subgenus of the genus *Carios* (Mans et al. 2019) or *Alectorobius* (Kneubehl et al. 2022), whereas another phylogenetic study considered *Antricola* as a valid genus (Mans et al. 2021). Because these phylogenetic studies lack the inclusion of the *Antricola* type species [*Antricola coprophilus* (McIntosh 1935)], herein we adopted the traditional classification in which *Antricola* is a valid genus (Nava et al. 2017), currently represented by 16 valid species (Muñoz-Leal et al. 2024).

The genus *Antricola* is restricted to the New World, where it has been reported on bats or from bat caves in southern United States, Mexico, Central America and Caribe, and the northern half of South America, including the Amazonian region (Guglielmone et al. 2003; Muñoz-Leal et al. 2024). *Antricola* is undoubtedly the most peculiar tick genus because it is the only one whose hematophagy is restricted solely to the larval stage, parasitizing bats (Černý 1969; De la Cruz 1973). The post-larval stages (nymphs and adults) are free-living (De la Cruz 1973), and there is evidence that they feed directly on the guano of insectivorous bats (De la Cruz 1992; Ribeiro et al. 2012; Oliveira et al. 2024). Moreover, *Antricola* ticks are strictly associated with hot caves, where colonies of thousands of insectivorous bats often live together, producing abundant guano that creates a noisome atmosphere rich in nitrogen compounds (ammoniated), high temperatures (30–38 °C) and relative humidity close to 100% (De la Cruz 1976; Cruz 1992; Labruna et al. 2011; Venzal et al. 2018). These peculiar habitats result in large abundance of *Antricola* ticks, especially over the guano (De la Cruz 1973; Cruz 1992; Oliveira et al. 2024). However, to the best of our knowledge, no concrete quantification of the population of *Antricola* in a hot cave has ever been reported.

In 2008, we reported two *Antricola* species - *Antricola guglielmonei* Estrada-Peña, Barros-Battesti & Venzal, , and *Antricola delacruzi* Estrada-Peña, Barros-Battesti & Venzal, 2004 - in a hot cave in the western Brazilian Amazon (Labruna et al. ). The ticks were found in a cave inhabited by an abundant population of insectivorous bats, natural hosts of abruna et al. *Antricola* spp. During subsequent visits to this cave for other studies (Nava et al. 2010; Labruna et al. 2011, 2021; Venzal et al. 2018), we always noticed large numbers of *Antricola* ticks walking across the abundant bat guano on the cave floor. In this study, we quantified tick samples from this cave and estimated the number of ticks wandering on the bat guano. Our results highlight the highest population and density of ticks ever recorded in a cave worldwide.

## Materials and methods

The cave in which the present study was performed is located within a primary Amazon Forest area in Porto Velho Municipality (08^o^40’S, 63^o^51’W), state of Rondônia, western Brazilian Amazon. It was discovered in 2003 by residents of Porto Velho. The cave´s rock formation is made up of laterite. Although it is not a cave for tourism or speleology, its surroundings have been progressively deforested. Because the cave is located within the Natural Park of Porto Velho, we will refer to this cave as the Natural Park Cave.

The following five argasid species have been reported in Natural Park Cave, all associated with insectivorous bats of the genus *Pteronotus* (Chiroptera: Mormoopidae): *A. guglielmonei*, *A. delacruzi*, *Nothoaspis amazoniensis* Nava, Venzal & Labruna, , *Ornithodoros rondoniensis* (Labruna et al. ), and *Ornithodoros marinkellei* Kohls, Clifford & Jones, 1969 (Labruna et al. ; 2011; Nava et al. ). During our previous visits to this cave along the last 20 years, there was always a large population of bats (possibly thousands of bats) and the floor was always covered by an abundant amount of fresh and humid guano. The latter, always densely populated with *Antricola* spp. ticks. On the other hand, the remaining three tick species (*N. amazoniensis*,* O. rondoniensis* and *O. marinkellei*) were collected mostly on the cave walls and ceiling, and only rarely on the guano (Labruna et al. ; 2011; Nava et al. 2010).

**Fig. 1:**
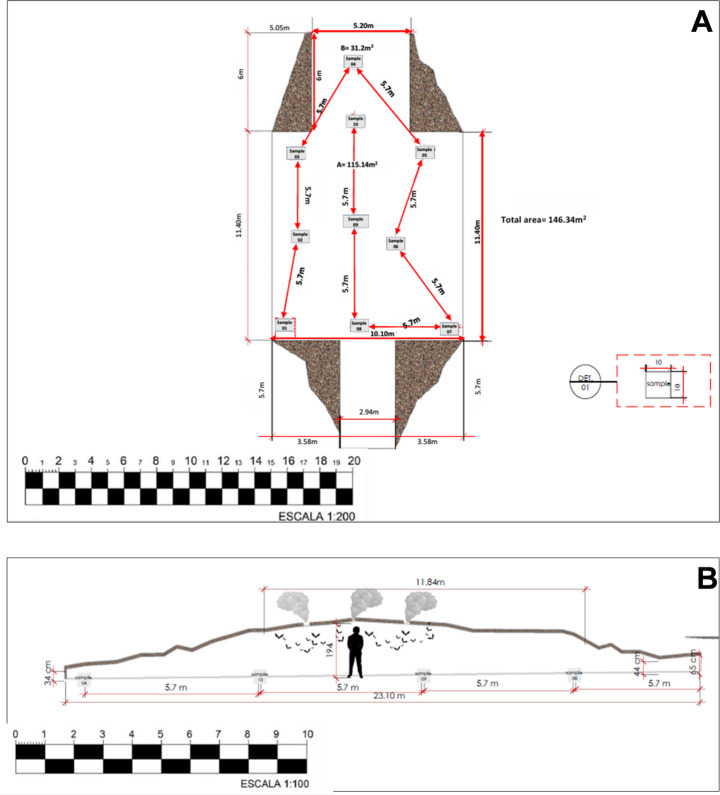
Drawn plan of the internal dimensions of the Natural Park Cave in Porto Velho Municipality, state of Rondônia, western Brazilian Amazon. (**A**) Floor plan of the cave, indicating the measurements of length and width of the internal cavity of the cave. The locations of all 10 points (samples 1 to 10) of collection of ticks in the guano are indicated. (**B**) Longitudinal plan of the cave, indicating the measurements of length (depth) and height in relation to the flat ground covered with guano. The locations of four central points (4, 10, 9, and 8) of collection of ticks in the guano are indicated. The cave’s internal height is 194 cm highest in the middle, lowest (34 cm) at the end, with an entrance height of 65 cm and a width of 2.94 m. Tick presence was recorded starting 5.70 m from the cave entrance. The main cave chamber covers 115.14 m², while the tunnel chamber at the end of the cave spans 31.2 m², totaling an area of 146.34 m² in which *Antricola* ticks were found on ground. Bats were absent on the cave floor but formed a large colony on the ceiling

During early 2024, we visited the Natural Park Cave with the aim of taking measurements and determining its width, length, depth, and height. As the cave appears to have a single chamber, the measurements were relatively simple, as shown in Fig. 1. Thereafter, the cave was visited twice for collection of tick samples; the first visit on July 21 (dry season), the second visit on December 9 (rainy season). In each visit, ticks were collected from 10 pre-determined and nearly equidistant points on the floor (bat guano), as illustrated in Fig. 1A. The 10 points were numbered from 1 to 10, each one consisting of an area of 100 cm^2^ of bat guano. For tick sampling, we used a 10 cm-square grid to determine an area of 100 cm² of guano at each of the 10 collection points. Using this square, guano containing ticks was collected to a depth of 2 cm (more than this was not necessary because ticks were not observed beyond 1 cm deep in the guano). The guano collected at each point was placed in a plastic bag containing 70% ethanol and previously identified with the collection point number. The bags were transported to the laboratory for quantification of the ticks. A total of 20 bags were collected, 10 from the dry season, 10 from the rainy season. Tick collections in this study have been previously authorized by the Brazilian environment official organ SISBio/IBAMA, under the permit no. 86648-1. During sampling collection, a digital thermo-hygrometer was left inside the cave (over the guano between points 9 and 10) and another outside the cave (next to the cave entrance), to obtain temperature and relative humidity (RH) values.

In the laboratory, the contents of each bag were entirely observed under a stereoscope microscope through several sequential aliquots within Petri dishes. All tick specimens were collected and placed in clean Petri dishes, where they were counted and identified according to species and developmental stages (larvae, nymphs, adult males and females). Tick species identifications followed morphological descriptions/redescriptions (Estrada-Peña et al. 2004; Labruna et al. 2008; 2011) and recent taxonomic keys (Muñoz-Leal et al. 2024).

Population density estimates were calculated using standard formulas in Microsoft Excel (Microsoft Corporation, Redmond, WA, USA). To estimate the total number of ticks on the cave floor (area covered by guano), the numbers of ticks present in the 10 samples of 100 cm² were added together to obtain the number of ticks in 1000 cm², which corresponds to 0.1 m². The value obtained was converted using a rule of three to the number of ticks per square meter (1 m^2^), which corresponded to the density of ticks on the cave floor. This density per square meter was multiplied by the total area covered by guano to estimate the total number of ticks on the cave floor. The collected tick specimens have been deposited in the Didactic and Scientific Collection of São Lucas University, Porto Velho, Rondônia.

## Results

Based on measurements taken inside the Natural Park Cave in early 2024, it was estimated that the cave floor, particularly the area covered with guano, has an area of 146.34 m² (Fig. 1). In this case, we considered the sum of the two internal areas covered by guano: a larger one measuring 115.14 m² (11.40 m long, 10.10 m wide) and a smaller one at the back of the cave measuring 31.20 m² (6.00 m long × 5.20 m wide) (Fig. 1A). The first 5.70 m from the cave entrance were excluded from this calculation because they corresponded to a section without guano and, therefore, without the presence of *Antricola* spp. During our sample collections in the mornings of July 21 (dry season) and December 9 (rainy season), mean temperatures inside the cave were 35.1 and 36.3 °C, respectively, contrasting to the external mean temperatures of 26.8 and 27.0 °C, respectively. Similarly, the RH mean values were also much higher inside (88–99%) than outside (68–75%) the cave (Table 1).

**Table 1:** Time and environmental data of the Natural Park Cave in Porto Velho, Rondônia, Brazil, during tick sample collections in the dry season and in the rainy season of 2024

Data	Dry season (21 July)	Rainy season (9 December)
Time of sample collections	From 8 to 11 am	From 8:30 to 11:30 am
External temperature	26.8Cº	27Cº
External humidity	68%	75%
Internal cave temperature	35.1Cº	36.3Cº
Internal cave humidity	88%	99%

**Fig. 2:**
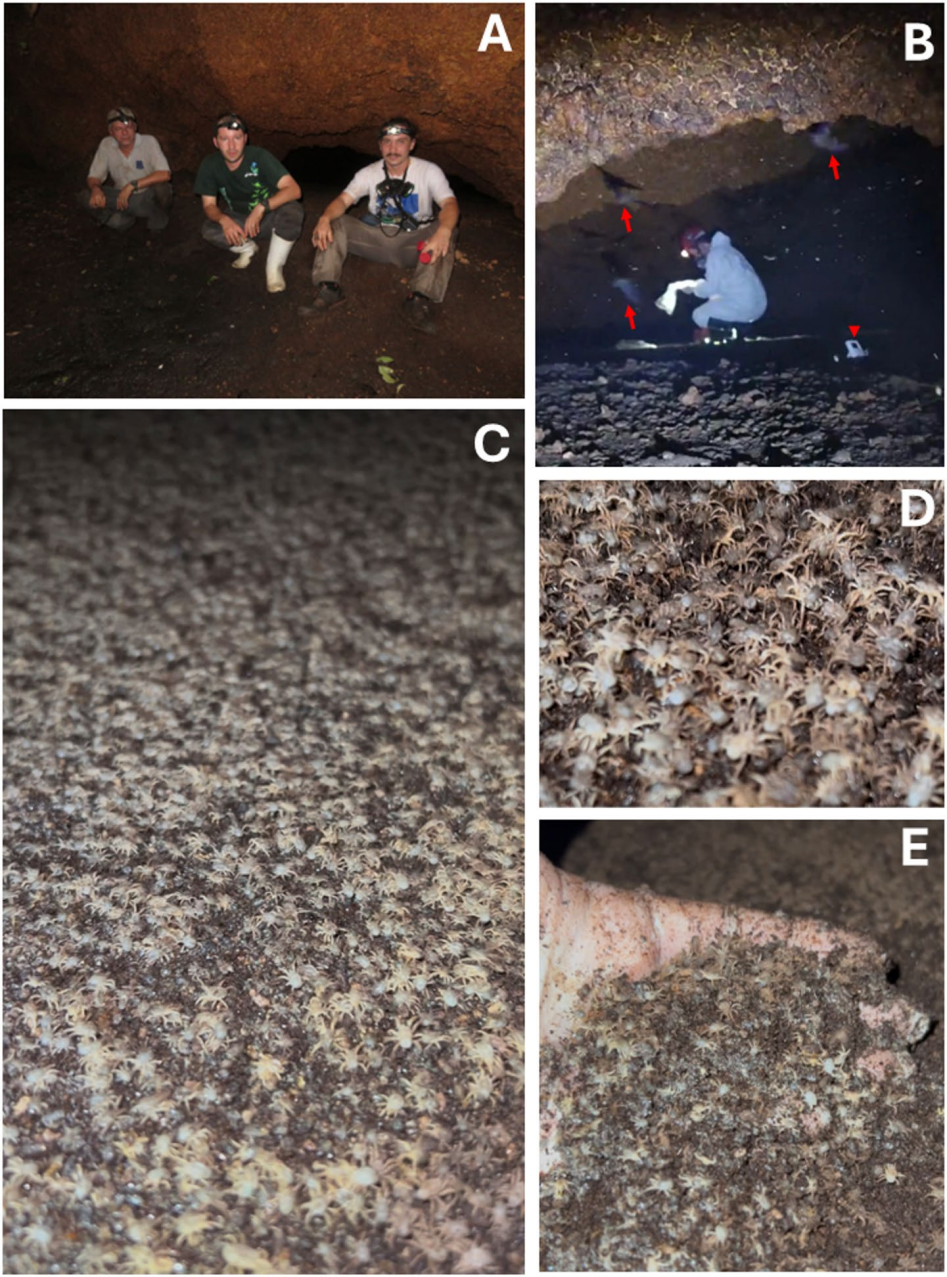
Photographs of the Natural Park Cave in Porto Velho Municipality, state of Rondônia, western Brazilian Amazon, when tick samples were collected in the guano. (**A**) External view of the cave entrance, where three researchers are seated, giving an idea of the low ceiling height at the cave entrance. (**B**) A researcher working in the cave during the dry season collection. Note flying bats (arrows) and a thermohydrometer on the guano (arrowhead). (**C**) Wide view of guano covered with *Antricola* ticks. (**D**) Closer view of guano covered with *Antricola* ticks. (**E**) Detail of ticks in guano after the researcher removed a portion of guano from the cave floor with his hand

During our visits to the cave in the dry (21 July 2024) and rainy (9 December 2024) seasons, the guano was always covered with ticks crawling around (Fig. 2, Supplemental Video S1). The numbers of collected ticks, according to species and developmental stage, in each of the 10 points of the guano in the dry and rainy seasons, are presented in Tables 2 and 3, respectively. A total of 7,388 and 8,753 ticks were collected in the dry and rainy seasons, respectively. In both seasons, > 99% of the collected ticks were of the genus *Antricola*, whose adults were nearly all identified as *A. delacruzi*; only a few adults of *A. guglielmonei* were identified. The *Antricola* spp. nymphs were retained at the genus level because it was not possible to separate them into the two species with certainty. A few specimens of other two species, *O. rondoniensis* and *O. marinkellei*, were also collected from the guano samples in both seasons, but they corresponded to < 1% of all collected ticks. Besides abundant ticks, other invertebrate animals were rarely seen in the guano samples; these included small beetles and dipteran larvae.

**Table 2:** Number of ticks collected in 10 samples of guano in the floor of the Natural Park Cave in the state of Rondônia, Brazil, on 21 July 2024 (dry season).

Dry season samples	Number of ticks according to species and stage					
	*Antricola* spp. nymphs	*Antricola delacruzi*	*Antricola guglielmonei*	*Ornithodoros rondoniensis*	*Ornithodoros marinkellei*	Total
1	240	15 F, 23 M			6 F, 4 M	288
2	444	23 F, 47 M			4 F, 4 M	522
3	897	16 F, 22 M			1 F	936
4	546	36 F, 52 M			2 M, 1 L	637
5	1070	22 F, 63 M	1 F, 1 M		2 F, 2 M	1161
6	583	29 F, 23 M			1 F, 3 M	639
7	1411	55 F, 64 M		2 N	5 F, 4 M, 2 L	1,543
8	222	16 F, 14 M	1 M		4 F, 2 M, 2 L	261
9	610	34 F, 44 M	1 M		2 F, 3 M, 1 L	695
10	631	23 F, 51 M			1 M	706
Total (1000 cm^2^)	6654	269 F, 403 M	1 F, 3 M	2 N	25 F, 25 M, 6 L	7,388
% of the total	90.06	9.10	0.05	0.03	0.76	100

Ticks were identified to genus and species and developmental stage (L: larvae; N: nymphs, M: adult males, and F: adult females). Each of the 10 samples represented the number of ticks in a 100 cm^2^ area of the guano surface

**Table 3:** Number of ticks collected in 10 samples of guano in the floor of the Natural Park Cave in the state of Rondônia, Brazil, on 9 December 2024 (rainy season)

Rainy season samples	Number of ticks according to species and stage					
	*Antricola* spp. nymphs	*Antricola delacruzi*	*Antricola guglielmonei*	*Ornithodoros rondoniensis*	*Ornithodoros marinkellei*	Total
1	722	12 F		1 N	5 F, 3 M	743
2	521	8 F, 1 M			1 L	531
3	896	6 F, 7 M		1 N	2 F, 2 L	914
4	492	2 F, 11 M		1 N	2 M, 1 N	509
5	1079	7 F, 6 M		1 N	1 M, 3 L	1,097
6	963	5 F, 1 M			2 M	971
7	812	7 F, 9 M			0	828
8	381	20 F, 12 M			0	413
9	974	51 F, 22 M		1 N	3 F, 1 M, 2 L	1,054
10	1667	9 F, 9 M		2 N	3 F, 1 M, 2 L	1,693
Total (1000 cm^2^)	8507	127 F, 78 M	0	7 N	13 F, 10 M, 1 N, 10 L	8,753
% of the total	97.19	2.34	0	0.08	0.39	100

Ticks were identified to genus and species and developmental stage (L: larvae; N: nymphs, M: adult males, and F: adult females). Each of the 10 samples represented the number of ticks in a 100 cm^2^ area of the guano surface

Since the number of ticks shown in Tables 2 and 3 were collected from a 1,000 cm^2^ area of the guano (sum of ten 100 cm^2^-points), they represent a density of 7,388 ticks/1,000 cm^2^ (in the dry season) and 8,753 ticks/1,000 cm^2^ (in the rainy season). After converting these numbers to square meters, the density of ticks in the cave floor is 73,880 ticks/m^2^ and 87,530 ticks/m^2^, respectively, with a mean of 80,705 ticks/m^2^ (Table 4). Considering the total area of the cave floor that was covered by guano (146.34 m^2^), the estimated number of ticks in the cave guano was 10,811,599 (dry season) and 12,809,140 (rainy season); mean: 11,810,370 ticks (Table 4).

**Table 4:** Quantitative data on ticks collected from bat guano on the floor of the Natural Park Cave in Rondônia State, Brazil, during the dry and rainy seasons of 2024

Tick quantitative data	Dry season	Rainy season	Mean or total
Mean No. ticks/100 cm^2^	738.8	875.3	807.1
Median	667	871	724.5
Standard Deviation	389.6	372.2	377.4
Range	261–1,543	413–1,693	261–1,693
95% Confidence Interval	460.1–1017.5	609.1–1141.5	630.5–983.7
Coefficient of Variation	52.7%	42.5%	46.8%
Variance	151,745.73	138,506.01	142,390.89
No. ticks collected in 1,000 cm^2^ (0.1 m^2^)	7388	8753	8070
Tick density (No. ticks/m^2^)	73,880	87,530	80,705
Estimated No. ticks in the cave*	10,811,599	12,809,140	11,810,370

*Calculated for an area of 146.34 m^2^, which corresponded to the cave’s internal floor that was covered by bat guano (see Fig. 1B)

## Discussion

The abundance of ticks (mean: 11,810,370 specimens) and mean density (80,705 ticks/m²) reported in the guano covering the floor of an Amazonian cave presented in our study, are unprecedented. Due to the insignificant proportion of ticks of the genus *Ornithodoros* (less than 1%) found in our samples, for practical purposes of discussion, we will consider that the values found refer to ticks of the genus *Antricola*.

All *Antricola* ticks collected on the guano were nymphs or adults. No larvae or eggs were found. This is possibly related to the fact that some *Antricola* females lay their eggs in cracks and crevices in cave walls, as previously reported for *A. coprophilus* in Mexico and the United States (Mazzotti 1940; Cooley and Kohls 1944). During our numerous previous visits to the Natural Park cave over the last 20 years, we always saw *Antricola* ticks climbing the walls, but in low numbers. We suspect that these were mostly females, as we once observed an *A. delacruzi* female laying eggs deep within wall crevice in the cave (F.A.T and M.B.L., unpublished data). This behavior would explain the absence of eggs and unfed larvae in our guano samples. On the other hand, the absence of engorged larvae (which may naturally detach from bats and therefore be on the cave floor) in our guano samples may be due to two factors: (i) the larvae constitute a very small portion of the *Antricola* population, and (ii) the pre-molting period of engorged larvae to N1 nymphs must be very rapid at high temperature conditions inside the caves. The hypothesis that the proportion of larvae is small is related to the fact that it is rare to find *Antricola* larvae parasitizing bats, even when the number of nymphs and adults in the guano is extremely high. This scenario was reported by Philip (1940) in Arizona, and by Černý and Dusbábek (1967) and De la Cruz (1992) in Cuba. It was also confirmed by Labruna et al. (2011) in the Natural Park Cave, where dozens of bats were examined for attached ticks, resulting in higher prevalence and abundance of *O. marinkellei* larvae than *Antricola* spp. larvae, even though the number of *O. marinkellei* adults found on the cave walls was extremely low (Labruna et al. 2011).

In this scenario, where the population of nymphs and adults far exceeds that of eggs and larvae, a plausible explanation would be the guanophagous behavior of *Antricola* nymphs and adults, as suggested by De la Cruz (1992). In a study in another hot cave in Brazil, we observed *Antricola* behaving as if they were feeding on fresh guano (Oliveira et al. 2024). In fact, the toothless spoon-like hypostome of the post-larval stages of *Antricola* is compatible with this feeding behavior (De la Cruz 1992; Oliveira et al. 2024). Finally, a transcriptome study of the salivary glands of *A. delacruzi* revealed that they are more compatible with guanophagia than with hematophagia (Ribeiro et al. 2012). That said, the longevity of *Antricola* nymphs and adults might be extremely long in the cave, given the constant supply of food (fresh guano). This would explain the high population density and abundance of these ticks on the guano in hot caves.

Given the dependence of the post-larval stages of *Antricola* spp. on bat guano, it was possible to estimate the population of these ticks in Natural Park Cave, since all the soil covered by guano could be accessed by representative samples. Thus, even though there was no quantification of ticks on the walls and ceiling or on the bats, and no eggs or larvae were counted, it is possible that our population estimate is very close to the actual number, given the factors explained above. Although there are no previous studies estimating the population of *Antricola* ticks in caves, some isolated data on population density have been reported. Over the guano surface in a hot cave in Arizona, USA, Philip (1940) reported that a bagged sample of guano from an estimated surface area of one square foot (= 0.093 square meter) contained 301 *A. coprophilus* ticks from early nymphs to adults. This density (301/0.093 m^2^) corresponds to 3,236.6 ticks/m^2^. For *A. marginatus* in a cave in Cuba, Černý and Dusbábek (1967) reported ≈ 2,000 specimens/m^2^ on the guano in a part of the cave. Even though these values were restricted to one sample from each cave, they are still much lower than the mean density of 80,714.6 ticks/m^2^ found in the present study. Due to the lack of data on this subject, we propose that the tick density observed in the Amazonian cave in this study is the highest ever recorded in a bat cave worldwide.

After studying *Antricola* ticks for decades in Cuba, De la Cruz (1992) stated the migration of bat communities between caves can significantly alter local species diversity, potentially reducing or eliminating parasite species such as ticks. Therefore, studies like this are essential to reveal little-known but ecologically significant host-parasite dynamics and may catalyze renewed efforts to protect subterranean ecosystems and their unique fauna. Indeed, ecological integrity of the Amazon Forest surrounding the Natural Park Cave plays a critical role in sustaining the stability of this exceptionally dense tick community. Habitat disruption caused by deforestation, wildfires, and other anthropogenic pressures in the vicinity of this protected area poses a severe threat to the long-term survival of both the bat hosts and their associated ectoparasites. Such environmental disturbances could trigger bat displacement, leading to the collapse of this highly specialized and localized tick assemblage. This study highlights the urgent need for reinforced surveillance and environmental governance. Conservation strategies must prioritize not only the protection of cave ecosystems but also the broader landscape connectivity that ensures host-parasite dynamics will remain undisturbed. The loss of this unique biological phenomenon would represent an irreplaceable gap in our understanding of host-parasite coevolution and cave ecology.

## Supplementary Information

Below is the link to the electronic supplementary material.

Supplementary Material 1: One of us (F.A.T.) entering the Natural Park Cave and showing the guano densely populated by moving *Antricola* ticks. The researcher is wearing a chemical mask due to the atmosphere inside the cave, which is rich in ammonia gases.

## Data Availability

All research data are described in the manuscript.
